# Prognostic value of the lactate-to-albumin ratio in critically ill chronic heart failure patients with sepsis: insights from a retrospective cohort study

**DOI:** 10.3389/fmed.2025.1593524

**Published:** 2025-07-15

**Authors:** Junqi Gou, Chaohui Liu, Mingjian Lang, Fengyou Yao

**Affiliations:** Department of Cardiology, Geriatric Diseases Institute of Chengdu/Cancer Prevention and Treatment Institute of Chengdu, Chengdu Fifth People’s Hospital (The Second Clinical Medical College, Affiliated Fifth People’s Hospital of Chengdu University of Traditional Chinese Medicine), Chengdu, China

**Keywords:** chronic heart failure, sepsis, lactate-to-albumin ratio, prognostic biomarker, retrospective cohort study

## Abstract

**Background and objectives:**

Critically ill patients with chronic heart failure (CHF) complicated with sepsis are associated with a high mortality risk. The lactate-to-albumin ratio (LAR) has been shown to correlate with poor prognosis in various critical illnesses. However, the relationship between LAR and the short-and long-term prognosis of critically ill patients with CHF and sepsis has not been thoroughly explored. Therefore, this study aimed to evaluate the prognostic value of LAR in critically ill patients with CHF and sepsis.

**Methods:**

A retrospective analysis was conducted on the clinical data of 2,416 ICU-managed critically ill patients with CHF and sepsis. Based on the optimal cutoff value, patients were divided into higher LAR and lower LAR groups. Multivariable Cox proportional hazards models were used to assess the association between LAR and all-cause mortality at different time points (ICU, in-hospital, 14-day, 28-day, and 90-day). Kaplan–Meier survival curves were used to evaluate the differences in all-cause mortality risk between the two groups. The receiver operating characteristic (ROC) curve is used to evaluate the predictive ability, sensitivity, specificity, and area under the curve (AUC) of LAR for predicting in-hospital mortality in patients with CHF and sepsis. Restricted cubic spline (RCS) analysis was performed to examine the potential dose–response relationship between LAR and all-cause mortality at each time point. Subgroup analyses further explored the impact of patient characteristics on the prognostic value of LAR.

**Results:**

LAR was significantly associated with ICU, in-hospital, 14-day, 28-day, and 90-day all-cause mortality. The higher LAR group had a higher risk of death compared to the lower LAR group (all *p* < 0.001). Cox regression analysis confirmed that LAR was an independent prognostic factor for ICU, in-hospital, 14-day, 28-day, and 90-day all-cause mortality in critically ill patients with CHF and sepsis. Kaplan–Meier survival curves further confirmed the significant association between LAR and poor prognosis. The ROC curve analysis shows that LAR has a better predictive value for the prognosis of patients with CHF and sepsis compared to lactate and albumin. RCS analysis demonstrated a linear relationship between LAR and ICU, in-hospital, 14-day, 28-day, and 90-day all-cause mortality. Subgroup analyses revealed consistent prognostic effects of LAR across different clinical subgroups, with no significant interaction observed.

**Conclusion:**

LAR is an independent predictor of short-term and long-term all-cause mortality in critically ill patients with CHF and sepsis. LAR has the potential to serve as a valuable prognostic biomarker in this population, providing significant implications for clinical decision-making and patient management.

## 1 Introduction

Chronic heart failure (CHF) is one of the leading causes of cardiovascular-related mortality worldwide, with persistently high incidence and death risk (1, 2). In recent years, hospitalizations and readmissions among CHF patients have significantly increased, thereby further increasing the healthcare burden (3). When CHF is complicated by sepsis, clinical outcomes deteriorate markedly, and the risk of mortality escalates dramatically (4–6). Due to prolonged cardiac dysfunction, reduced perfusion, and inadequate organ perfusion, CHF patients not only suffer from metabolic disturbances and tissue hypoxia but also exhibit immunosuppression (7). This impairment in immune function is closely associated with the structural and functional damage of the heart, and may be further exacerbated by the long-term use of medications such as diuretics, angiotensin-converting enzyme (ACE) inhibitors, and β-blockers, thereby diminishing the body’s ability to combat infections (8–10).

In addition, CHF patients often experience fluid retention and alterations in vascular permeability, factors that facilitate pathogen invasion (11, 12). It is noteworthy that in the ICU setting, patients frequently undergo invasive procedures such as vascular catheterization and mechanical ventilation, and are commonly treated with broad-spectrum antibiotics, corticosteroids, and renal replacement therapy. These interventions can disrupt the normal microbial flora and further suppress immune responses (13). Consequently, CHF patients are not only predisposed to infections, but also tend to experience rapid disease progression that may culminate in sepsis (14, 15). Despite advances in ICU management, both short-and long-term mortality rates remain high in this population, underscoring the urgent need for novel biomarkers to enable earlier and more accurate prognostic assessments to guide clinical decision-making.

The lactate-to-albumin ratio (LAR) has emerged as a promising biomarker, demonstrating a strong association with adverse outcomes in various critically ill populations (16–19). Lactate, a byproduct of metabolic dysregulation and tissue hypoxia, typically correlates positively with systemic inflammatory responses, organ failure, and mortality risk (20, 21). Moreover, albumin serves as an important indicator of nutritional status, hepatic function, and systemic inflammation, with its decline often signifying greater disease severity (22, 23). Recent studies have shown that elevated LAR is closely linked to poor prognosis in several critical illness scenarios, such as acute kidney injury, liver cirrhosis, acute pancreatitis; however, its prognostic value in CHF patients with sepsis remains to be fully elucidated.

Therefore, the present study aims to evaluate the prognostic significance of LAR in critically ill CHF patients with sepsis. We retrospectively analyzed clinical data from 2,416 ICU-admitted patients with CHF complicated by sepsis, investigating the association between LAR and all-cause mortality at various time points, including ICU, in-hospital, 14-day, 28-day, and 90-day all-cause mortality. Additionally, this study will further delineate the high-risk characteristics of this patient population and explore the potential of LAR as an independent prognostic marker for clinical application. Through this study, we hope to provide a novel biomarker to facilitate risk stratification and individualized treatment in this high-risk patient group.

## 2 Methods

### 2.1 Data source and study population

This retrospective cohort study utilized data from Medical Information Market for Intensive Care IV (MIMIC-IV, version 3.0), a publicly available critical care database developed by the Massachusetts Institute of Technology Laboratory for Computational Physiology (24). MIMIC-IV contains comprehensive de-identified electronic health records of patients admitted to the Beth Israel Deaconess Medical Center in Boston, Massachusetts, between 2008 and 2022. The database includes demographic information, vital signs, laboratory results, medication records, and clinical diagnoses, making it a valuable resource for large-scale epidemiological and prognostic studies. To ensure data security and ethical compliance, the research team completed the Collaborative Institutional Training Initiative program and passed the necessary examination before being granted access to the MIMIC-IV (version 3.0).

**Figure 1:**
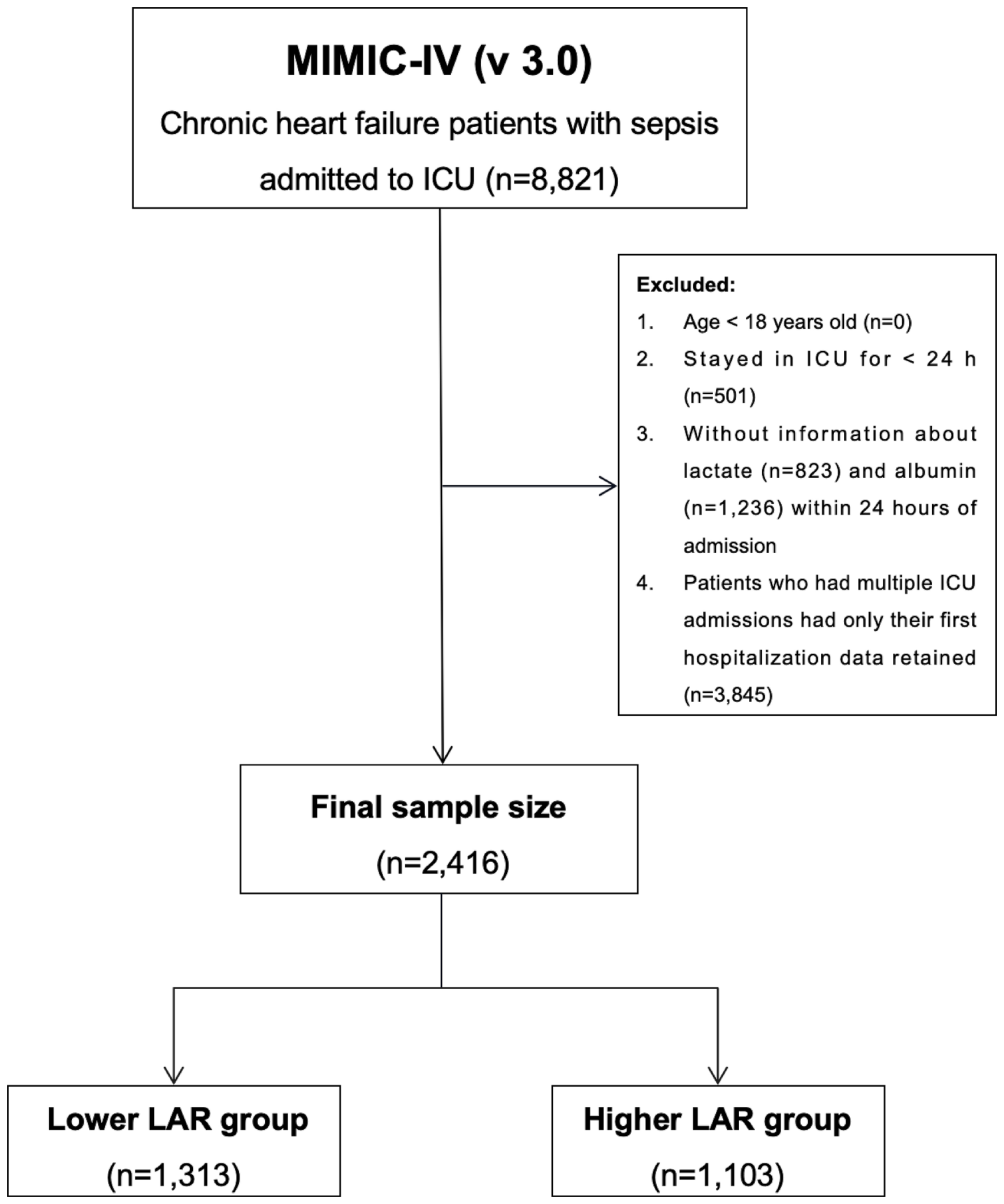
Flowchart for participants from MIMIC-IV (v 3.0).

Patients included in this study met the following inclusion criteria: (1) age ≥18 years; (2) diagnosis of CHF as identified by International Classification of Diseases, 9th and 10th Revision (ICD-9/10) codes, with specific diagnostic criteria of ICD-9 (42822, 42823, 42832, 42833, 42842, 42843) and ICD-10 (I5022, I5023, I5032, I5033, I5042, I5043, I50812, I50813); (3) meeting Sepsis-3.0 definition, which includes suspected infection and a Sequential Organ Failure Assessment (SOFA) score ≥ 2 points (25). Exclusion criteria included: (1) ICU length of stay < 24 h; (2) absence of recorded serum lactate or albumin levels within the first 24 h of admission; (3) patients with multiple ICU admissions, with only the first ICU stay being considered. A total of 2,416 critically ill patients with CHF and sepsis who met the inclusion criteria were included in the final analysis (Figure 1). LAR was calculated using the lactate and albumin levels measured within the first 24 h of patient admission.

Importantly, all mortality outcomes (ICU, in-hospital, 14-day, 28-day, and 90-day) were defined as all-cause mortality, as specific cause-of-death information is not provided in the MIMIC-IV database.

### 2.2 Data extraction

Data extraction was performed using PostgreSQL (PostgreSQL Global Development Group, 2024) and Navicat Premium software (CyberTech Ltd., Hong Kong SAR), utilizing SQL for efficient retrieval of variables relevant to the study population.

The extracted variables included demographic characteristics such as age, sex, and ethnicity; clinical parameters including vital signs (e.g., heart rate, systolic and diastolic blood pressure, respiratory rate, body temperature); chronic comorbidities such as hypertension and diabetes mellitus; as well as acute clinical conditions reflecting illness severity at ICU admission, including respiratory failure and septic shock. Laboratory test results were also collected, including complete blood count, liver function, renal function, electrolytes, coagulation function, and arterial blood gas analysis. Additionally, treatment and intervention data were extracted, such as the use of vasopressors, mechanical ventilation, and continuous renal replacement therapy. Survival data, including ICU all-cause mortality, in-hospital all-cause mortality, and 14-day, 28-day, and 90-day all-cause mortality rates, were collected to assess both short-term and long-term patient outcomes.

All extracted variables were checked for completeness and accuracy before data analysis. The specific covariates extracted are detailed in Table 1.

**Table 1:** Covariates extracted in detail.

Items	Composition
Demographic variables	Age, Sex, Ethnicity
Comorbidities	Hypertension, Diabetes mellitus, Renal disease, Liver disease, Chronic obstructive pulmonary disease, Pneumonia, Asthma, Unstable angina pectoris, Myocardial infarction, Acute respiratory distress syndrome, Cerebral infarction, Malignancy, Septic shock, Respiratory failure
Vital Signs	Heart rate, Systolic blood pressure, Diastolic blood pressure, Mean arterial pressure, Respiratory rate, SPO_2_, Body temperature
Laboratory parameters	Neutrophil cells, Lymphocyte cells, Red blood cells, White blood cells, Erythrocyte distribution width, Platelet, Hemoglobin, Lymphocyte percentage, Hematocrit, Creatinine, Blood urea nitrogen, Albumin, Total bilirubin, Direct bilirubin, Aspartate aminotransferase, Alanine aminotransferase, Glucose, Triglyceride, Total cholesterol, High density lipoprotein cholesterol, Low density lipoprotein cholesterol, Prothrombin time, International normalized ratio, Potassium, Sodium, Calcium, Anion gap, Lactate, PH_2_, PCO_2_, PaO_2_
Clinical Treatments	Urinary catheter, Vasopressin, Ventilation, Continuous Renal Replacement Therapy, Norepinephrine
Drugs	Antiplatelet drugs, Lipid regulating drugs, Diuretics, Antibacterial drugs, Vasodilator drug, Anti-arrhythmic drug
Clinical Outcomes	ICU mortality, In-hospital mortality, 14-day mortality, 28-day mortality, 90-day mortality

### 2.3 Handling of outliers and missing data

To ensure the reliability and accuracy of the analysis, rigorous methods were applied to handle outliers and missing data. Outliers were addressed using the winsor2 command in STATA software, employing a winsorization method with 1 and 99% percentile cutoff points to limit extreme values in continuous variables. This approach helps preserve the overall trends in the data while minimizing the impact of potential outliers, thus reducing bias in the results.

For missing data, we first assessed the missing data rates for each variable. Variables with missing rates exceeding 15% were excluded from the analysis to avoid potential bias from excessive missing data. This exclusion criterion was implemented to ensure the robustness of the results and to minimize the impact of variables with substantial missing data, which might otherwise lead to biased or unreliable conclusions. For variables with missing rates ≤ 15%, we used multiple imputations to handle the missing values. This method generates multiple plausible estimates for missing data, thereby reducing bias and improving the stability and credibility of the analysis.

Through this approach, we ensured that only variables with a manageable level of missing data were included in the final analysis, and the imputation method enhanced the completeness of the dataset. By applying these procedures, we were able to maintain data integrity and enhance the robustness and reliability of the analytical results.

### 2.4 Clinical outcomes

The outcomes of this study included ICU all-cause mortality, in-hospital all-cause mortality, and all-cause mortality within 14 days, 28 days, and 90 days of admission. All mortality data were defined as death from any cause and were obtained from the MIMIC-IV database. All outcomes were based on clinical records and were verified multiple times to ensure the accuracy and completeness of the data.

### 2.5 Ethics statement

This study utilized data from a publicly available database, with all records de-identified to ensure patient privacy. Patient identities were replaced with random codes, thus informed consent was not required. The study adhered to the ethical principles outlined in the Declaration of Helsinki and was approved by the Institutional Review Board of the MIMIC-IV. The use of data complied with all relevant regulations for data access and ethical conduct, ensuring the transparency and integrity of the research.

### 2.6 Statistical analysis

Statistical analyses were performed using R software (version 4.2.2; R Foundation for Statistical Computing, Vienna, Austria), STATA software (version 16.0; StataCorp LLC, College Station, TX, USA), and IBM SPSS Statistics for Windows, version 22.0 (IBM Corp., Armonk, NY, USA).

For continuous variables with a normal distribution, data were expressed as mean ± standard deviation, and comparisons between groups were conducted using the t-test or analysis of variance. For continuous variables with a non-normal distribution, data were presented as median (interquartile range), and intergroup comparisons were performed using the Mann–Whitney U test or Kruskal-Wallis test. Categorical variables were presented as numbers and percentages, with differences between groups analyzed using the chi-square test or Fisher’s exact test.

**Figure 2:**
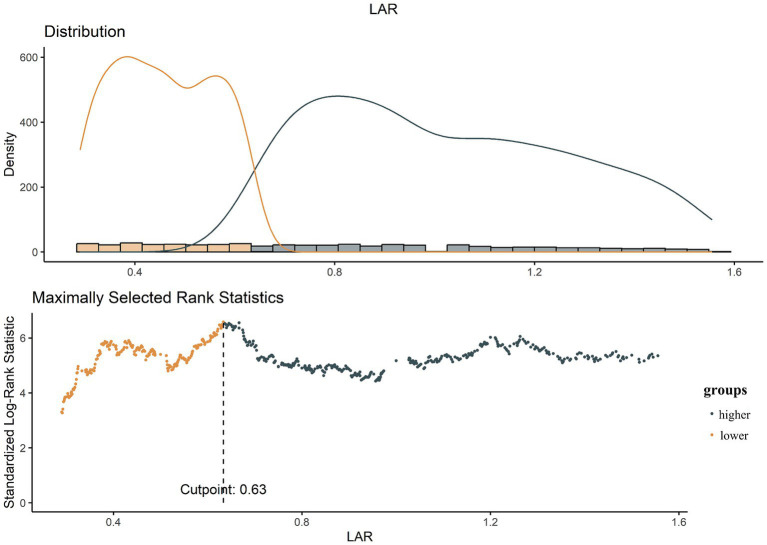
The choice of the optimal cutoff point maximized the risk ratio and the relationship between LAR ≥ 0.63 and the distribution of LAR.

The optimal cutoff value for LAR was determined using R software. Based on this cutoff, patients were categorized into a lower LAR group and a higher LAR group (Figure 2). To assess the association between LAR and all-cause mortality risk, multivariate Cox proportional hazards models were constructed to calculate hazard ratios (HRs) with 95% confidence intervals (CIs). Three models were developed for this analysis: Model 1 was the unadjusted baseline model; Model 2 adjusted for age, sex, and ethnicity; and Model 3 further adjusted for potential confounders, including body mass index, serum creatinine, white blood cell count, platelet count, hypertension, heart failure, respiratory failure, diabetes, use of vasopressors, continuous renal replacement therapy, and the SOFA.

Receiver operating characteristic (ROC) analysis was used to evaluate the predictive ability of LAR, lactate, and albumin at different time points for all-cause mortality risk in patients with CHF and sepsis, and to calculate the area under the curve (AUC).

Restricted cubic splines (RCS) were employed to explore the dose–response relationship between LAR and ICU, in-hospital, and 14-day, 28-day, and 90-day all-cause mortality. RCS was chosen because it allows for the modeling of non-linear relationships, which is essential in capturing more complex associations that linear models may miss. This method enables us to explore the potential non-linear dose–response relationship between LAR and mortality risk, providing a more accurate representation of the underlying data. The choice of knots in the RCS model was based on percentiles of the LAR distribution, which is a commonly used method to avoid overfitting and ensure model stability. We performed sensitivity analysis to assess the robustness of the results with different numbers of knots, and found that using four knots provided stable and consistent results.

Kaplan–Meier survival curves were generated to assess the survival probabilities of the lower and higher LAR groups for ICU all-cause mortality, in-hospital all-cause mortality, and all-cause mortality at 14 days, 28 days, and 90 days post-admission. The log-rank test was used to compare survival differences between the groups. Furthermore, a subgroup analysis was conducted to assess the consistency of the association between LAR and mortality across different subgroups stratified by age, sex, hypertension, diabetes, and respiratory failure.

All tests were two-sided, and a *p*-value of less than 0.05 was considered statistically significant.

## 3 Results

### 3.1 Baseline characteristics of participants

A total of 2,416 critically ill patients with CHF and sepsis were included in this study, with 1,414 (58.53%) male patients. Based on the determined optimal cutoff, 1,313 patients were categorized into the lower LAR group, and 1,103 patients into the higher LAR group. Significant differences in age were observed between the two groups, with patients in the lower LAR group being younger (73 [63–81] years) compared to those in the higher LAR group (74 [64–81] years; *p* = 0.03). Compared to the lower LAR group, patients in the higher LAR group had a faster heart rate, lower body temperature, and lower blood pressure (all *p* < 0.001).

Additionally, the higher LAR group had a significantly higher likelihood of having septic shock, and a higher frequency of norepinephrine use (all *p* < 0.05). There were also notable differences between the two groups in laboratory results, such as neutrophil counts, serum creatinine, albumin, aspartate aminotransferase, alanine aminotransferase, prothrombin time, potassium, and anion gap, among others (all *p* < 0.05). Furthermore, the higher LAR group had a higher frequency of meropenem, metronidazole, and vancomycin usage (all *p* < 0.05).

Regarding clinical outcomes, patients in the higher LAR group exhibited significantly higher ICU all-cause mortality, in-hospital all-cause mortality, and 14-day, 28-day, and 90-day all-cause mortality rates (all *p* < 0.05). More detailed information is presented in Table 2.

**Table 2:** Participant baseline characteristics.

Variable	Overall (*n* = 2,416)	Lower LAR (*n* = 1,313)	Higher LAR (*n* = 1,103)	*p* value
LAR	0.59 (0.40–0.95)	0.41 (0.32–0.51)	1.00 (0.77–1.44)	<0.001
Demographics				
Age, years	73 (63–81)	73 (63–81)	74 (64–81)	0.03
Men, *n* (%)	1,414 (58.53)	764 (58.19)	650 (58.93)	0.71
Ethnicity, *n* (%)				
Asian populations	64 (2.65)	26 (1.98)	38 (3.44)	0.002
White populations	1,539 (63.70)	877 (66.79)	662 (60.02)	
Black populations	237 (9.81)	114 (8.68)	123 (11.15)	
Others	576 (23.84)	296 (22.54)	280 (25.39)	
Comorbidities				
Hypertension, *n* (%)	835 (34.56)	475 (36.18)	360 (32.64)	0.07
Diabetes mellitus, *n* (%)	1,159 (47.97)	616 (46.92)	543 (49.23)	0.26
Renal disease, *n* (%)	2,276 (94.21)	1,223 (93.15)	1,053 (95.47)	0.02
Liver disease, *n* (%)	491 (20.32)	218 (16.60)	273 (24.75)	<0.001
Chronic obstructive pulmonary disease, *n* (%)	394 (16.31)	237 (18.05)	157 (14.23)	0.01
Pneumonia, *n* (%)	1,027 (42.51)	593 (45.16)	434 (39.35)	0.004
Asthma, *n* (%)	188 (7.78)	111 (8.45)	77 (6.98)	0.18
Unstable angina pectoris, *n* (%)	16 (0.66)	10 (0.76)	6 (0.544)	0.51
Myocardial infarction, *n* (%)	42 (1.74)	21 (1.60)	21 (1.90)	0.57
Acute respiratory distress syndrome, *n* (%)	38 (1.57)	22 (1.68)	16 (1.45)	0.66
Cerebral infarction, *n* (%)	378 (15.65)	205 (15.61)	173 (15.68)	0.96
Malignancy, *n* (%)	504 (20.86)	285 (21.71)	219 (19.85)	0.26
Septic shock, *n* (%)	707 (29.26)	285 (21.71)	422 (38.26)	<0.001
Respiratory failure, *n* (%)	1,453 (60.14)	789 (60.09)	664 (60.20)	0.96
Vital signs				
Heart rate, beats/min	88 (76–104)	87 (74–100)	91 (77–109)	<0.001
Systolic blood pressure, mmHg	115 (101–134)	119 (103–136.5)	113 (98–130)	<0.001
Diastolic blood pressure, mmHg	64 (53–76)	65 (55–77)	62 (51–75)	<0.001
Mean arterial pressure, mmHg	82 (70.67–94.33)	83.67 (72.33–95.67)	79 (68.33–92)	<0.001
Respiratory rate, times/min	20 (16–24)	20 (16–24)	20 (16–25)	0.10
SPO_2_, %	97 (94–100)	97 (94–100)	98 (94–100)	0.02
Body temperature, °C	36.72 (36.39–37.11)	36.72 (36.39–37.11)	36.67 (36.39–37.06)	<0.001
Laboratory parameters				
Neutrophil cells, 10^9^/L	7.59 (4.98–12.15)	7.3 (4.9–11.02)	8.08 (5.04–13.62)	0.001
Lymphocyte cells, 10^9^/L	1.11 (0.69–1.69)	1.06 (0.68–1.65)	1.16 (0.72–1.75)	0.05
Red blood cells, 10^9^/L	12 (8.5–16.9)	11 (7.9–15)	13.6 (9.3–19.1)	<0.001
White blood cells, 10^9^/L	3.41 (2.89–4)	3.39 (2.91–3.99)	3.43 (2.85–4.01)	0.72
Erythrocyte distribution width, %	15.5 (14.3–17.2)	15.4 (14.2–17.1)	15.7 (14.4–17.5)	<0.001
Platelets, 10^9^/L	185 (131–256)	192 (137–262)	177 (125–248)	<0.001
Hemoglobin, g/L	10.1 (8.5–11.7)	10 (8.5–11.7)	10.1 (8.4–11.8)	0.88
Lymphocyte percentage, %	8.3 (4.6–13.9)	8.7 (5.2–14.1)	7.6 (4–13)	<0.001
Hematocrit, %	31.4 (26.65–36.4)	31.4 (26.8–36.2)	31.5 (26.4–36.6)	0.83
Creatinine, mg/dL	1.5 (1–2.4)	1.4 (1–2.4)	1.6 (1.1–2.5)	<0.001
Blood urea nitrogen, mg/dL	33 (21–53)	32 (21–53)	35 (22–54)	0.08
Albumin, g/dL	3 (2.6–3.4)	3.2 (2.8–3.5)	2.8 (2.4–3.2)	<0.001
Total bilirubin, mg/dL	0.7 (0.4–1.3)	0.6 (0.4–1)	0.9 (0.5–1.7)	<0.001
Direct bilirubin, mg/dL	0.8 (0.3–1.9)	0.6 (0.2–1.5)	1.1 (0.4–2.4)	<0.001
Aspartate aminotransferase, U/L	41 (24–94)	35 (22–67)	51 (28–155)	<0.001
Alanine aminotransferase, U/L	26 (15–61)	23 (14–44)	32 (16–102)	<0.001
Glucose, mg/dL	137 (109–182)	133 (107–171)	145 (112–196)	<0.001
Triglyceride, mg/dL	126 (89–195.5)	122 (91–197)	129 (88–195)	0.84
Total cholesterol, mg/dL	147 (116–183)	148 (118.5–183)	146 (110–181)	0.25
High density lipoprotein cholesterol, mg/dL	43 (33–56)	45 (34–56)	42 (32–55)	0.04
Low density lipoprotein cholesterol, mg/dL	77 (53–102)	77 (55–102)	76 (50–102)	0.49
Prothrombin time, s	15.5 (13.4–20)	14.8 (13–18.3)	16.6 (14.1–21.65)	<0.001
International normalized ratio	1.4 (1.2–1.8)	1.3 (1.2–1.7)	1.5 (1.3–2)	<0.001
Potassium, mmol/L	4.3 (3.8–4.8)	4.2 (3.8–4.7)	4.4 (3.9–5)	<0.001
Sodium, mmol/L	138 (135–141)	138 (135–141)	138 (134–141)	0.02
Calcium, mg/dL	8.4 (7.9–8.8)	8.4 (7.9–8.9)	8.3 (7.7–8.8)	<0.001
Anion gap, mmol/L	15 (12–18)	14 (12–17)	16 (13–20)	<0.001
Lactate, mmol/L	1.7 (1.2–2.7)	1.3 (1–1.6)	2.9 (2.2–4)	<0.001
PH	7.36 (7.29–7.42)	7.37 (7.31–7.42)	7.35 (7.28–7.41)	<0.001
PCO_2_	42 (36–50)	43 (37–52)	41 (34–48)	<0.001
PaO_2_	81 (47–160.5)	83 (50–150)	79 (43–175)	0.12
Treatments				
Urinary catheter, *n* (%)	712 (29.47)	355 (27.04)	357 (32.37)	0.004
Vasopressin, *n* (%)	424 (17.55)	158 (12.03)	266 (24.12)	<0.001
Ventilation, *n* (%)	2,323 (96.15)	1,263 (96.19)	1,060 (96.10)	0.91
Continuous Renal Replacement Therapy, *n* (%)	364 (15.07)	169 (12.87)	195 (17.68)	0.001
Norepinephrine, *n* (%)	1,079 (44.66)	492 (37.47)	587 (53.22)	<0.001
Drugs				
Antiplatelet drugs				
Clopidogrel, *n* (%)	417 (17.26)	224 (17.06)	193 (17.50)	0.78
Aspirin *n* (%)	1,612 (66.72)	881 (67.10)	731 (66.27)	0.67
Lipid regulating drugs				
Atorvastatin, *n* (%)	1,063 (44.00)	597 (45.47)	466 (42.25)	0.11
Simvastatin, *n* (%)	326 (13.49)	204 (15.54)	122 (11.06)	0.001
Diuretics				
Spironolactone, *n* (%)	241 (9.98)	128 (9.75)	113 (10.24)	0.68
Furosemide, *n* (%)	2,085 (86.30)	1,147 (87.36)	938 (85.04)	0.10
Antibacterial drugs				
Amoxicillin-clavulanic acid, *n* (%)	67 (2.77)	40 (3.05)	27 (2.45)	0.37
Ampicillin sodium, *n* (%)	82 (3.39)	49 (3.73)	33 (2.99)	0.32
Piperacillin tazobactam, *n* (%)	735 (30.42)	364 (27.72)	371 (33.64)	0.002
Cefazolin, *n* (%)	243 (10.06)	127 (9.67)	116 (10.52)	0.49
Ceftazidime, *n* (%)	249 (10.31)	135 (10.28)	114 (10.34)	0.96
Ceftriaxone, *n* (%)	277 (11.47)	167 (12.72)	110 (9.97)	0.03
Meropenem, *n* (%)	407 (16.85)	186 (14.17)	221 (20.04)	<0.001
Imipenem, *n* (%)	10 (0.41)	5 (0.38)	5 (0.45)	0.78
Azithromycin, *n* (%)	441 (18.25)	269 (20.49)	172 (15.59)	0.002
Erythromycin, *n* (%)	80 (3.31)	41 (3.12)	39 (3.54)	0.57
Ciprofloxacin, *n* (%)	479 (19.83)	246 (18.74)	233 (21.12)	0.14
Levofloxacin, *n* (%)	311 (12.87)	183 (13.94)	128 (11.60)	0.09
Metronidazole, *n* (%)	456 (18.87)	188 (14.32)	268 (24.30)	<0.001
Vancomycin, *n* (%)	2,026 (83.86)	1,056 (80.43)	970 (87.94)	<0.001
Vasodilator drug				
Nitroglycerin, *n* (%)	636 (26.32)	337 (25.67)	299 (27.11)	0.42
Anti-arrhythmic drug				
Amiodarone, *n* (%)	676 (27.98)	310 (23.61)	366 (33.18)	<0.001
Clinical outcomes				
ICU mortality, *n* (%)	406 (16.80)	159 (12.11)	247 (22.39)	<0.001
In-hospital mortality, *n* (%)	605 (25.04)	250 (19.04)	355 (32.18)	<0.001
14-day mortality, *n* (%)	427 (17.67)	181 (13.79)	246 (22.30)	<0.001
28-day mortality, *n* (%)	644 (26.66)	281 (21.40)	363 (32.91)	<0.001
90-day mortality, *n* (%)	946 (39.16)	435 (33.13)	511 (46.33)	<0.001

### 3.2 Cox regression analysis results

As shown in Table 3, we constructed three Cox proportional hazard regression models to assess the impact of LAR on mortality at different time points.

**Table 3:** Cox proportional hazard ratios (HR) for all-cause mortality.

	Model 1		Model 2		Model 3	
	HR (95% CI)	*p* value	HR (95% CI)	*p* value	HR (95% CI)	*p* value
ICU mortality						
LAR (continuous)	1.24 (1.14–1.34)	<0.001	1.26 (1.17–1.37)	<0.001	1.21 (1.11–1.32)	<0.001
Lower LAR	Reference		Reference		Reference	
Higher LAR	1.75 (1.43–2.14)	<0.001	1.75 (1.43–2.14)	<0.001	1.55 (1.27–1.91)	<0.001
In-hospital mortality						
LAR (continuous)	1.20 (1.13–1.28)	<0.001	1.23 (1.15–1.31)	<0.001	1.14 (1.06–1.23)	<0.001
Lower LAR	Reference		Reference		Reference	
Higher LAR	1.68 (1.43–1.98)	<0.001	1.67 (1.42–1.96)	<0.001	1.49 (1.26–1.77)	<0.001
14-day mortality						
LAR (continuous)	1.26 (1.17–1.37)	<0.001	1.29 (1.19–1.40)	<0.001	1.14 (1.04–1.26)	0.004
Lower LAR	Reference		Reference		Reference	
Higher LAR	1.71 (1.41–2.08)	<0.001	1.67 (1.38–2.03)	<0.001	1.44 (1.18–1.76)	<0.001
28-day mortality						
LAR (continuous)	1.25 (1.17–1.34)	<0.001	1.28 (1.19–1.37)	<0.001	1.16 (1.07–1.25)	<0.001
Lower LAR	Reference		Reference		Reference	
Higher LAR	1.67 (1.43–1.95)	<0.001	1.62 (1.38–1.89)	<0.001	1.44 (1.23–1.70)	<0.001
90-day mortality						
LAR (continuous)	1.26 (1.19–1.33)	<0.001	1.28 (1.20–1.36)	<0.001	1.16 (1.09–1.24)	<0.001
Lower LAR	Reference		Reference		Reference	
Higher LAR	1.57 (1.38–1.79)	<0.001	1.53 (1.34–1.74)	<0.001	1.37 (1.20–1.57)	<0.001

Model 1: Unadjusted.

Model 2: Adjusted age, gender, and ethnicity.

Model 3: Adjusted age, gender, ethnicity, body mass index, serum creatinine, white blood cells, platelet, hypertension, heart failure, respiratory failure, diabetes, vasopressin, continuous renal replacement therapy, and Sequential Organ Failure Assessment.

In the unadjusted model (Model 1), for each 1-unit increase in LAR, the risks of ICU all-cause mortality, in-hospital all-cause mortality, and all-cause mortality at 14, 28, and 90 days were significantly higher (for example, ICU mortality HR: 1.24, 95% CI: 1.14–1.34, *p* < 0.001). When patients were categorized into lower and higher LAR groups, the higher LAR group exhibited significantly higher mortality risks at each of the above endpoints compared to the lower LAR group (all *p* < 0.001). In the model adjusted for age, sex, and ethnicity (Model 2), the associations were somewhat attenuated but still statistically significant.

In Model 3, which adjusted for additional clinical and laboratory parameters, the association between LAR and mortality at each time point remained significant. For ICU all-cause mortality, for example, each 1-unit increase in LAR was associated with a 21% increase in the risk of death (HR: 1.21, 95% CI: 1.11–1.32, *p* < 0.001), while the risk of death in the higher LAR group compared to the lower LAR group increased by 55% (HR: 1.55, 95% CI: 1.27–1.91, *p* < 0.001). Similarly, for in-hospital mortality, 14-day, 28-day, and 90-day mortality, LAR remained an independent adverse prognostic factor in Model 3, and its hazard ratios remained statistically significant after adjustment (all *p* < 0.05).

Therefore, whether measured as a continuous variable or as a categorical grouping, LAR is significantly associated with both short-term and long-term all-cause mortality risk in critically ill patients with CHF and sepsis. Even after adjusting for potential confounders, higher LAR remains an important independent predictor of poor short-term and long-term prognosis in these patients.

### 3.3 Kaplan–Meier survival analysis results

**Figure 3:**
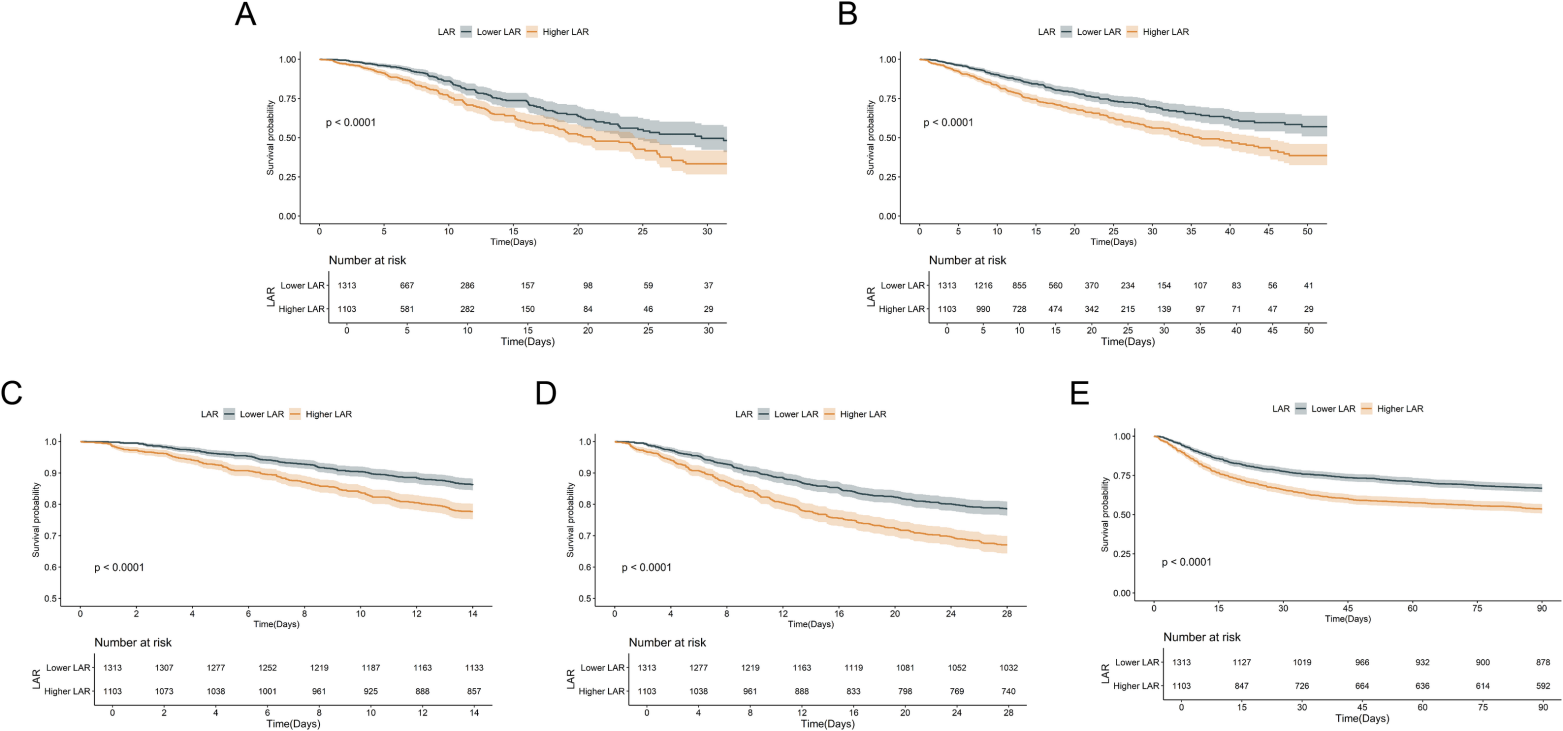
Kaplan–Meier survival analysis curves for all-cause mortality. Kaplan–Meier curves and cumulative incidence of ICU **(A)**, In-hospital **(B)**, 14-day **(C)**, 28-day **(D)**, and 90-day **(E)** all-cause mortality stratified by LAR groups.

Figure 3 illustrates the cumulative survival curves of patients in both groups at different time points. At all observed time points, the survival rate of the higher LAR group was significantly lower than that of the lower LAR group, and the difference was statistically significant (Log-rank test, *p* < 0.0001). The survival curves show that, over time, the survival gap between the two groups gradually widened, suggesting that higher LAR levels are closely associated with poorer short-term and long-term outcomes. This finding is consistent with the results of the Cox regression analysis, further validating LAR as an important prognostic indicator of poor outcomes in critically ill patients with CHF and sepsis.

### 3.4 Predictive efficacy of LAR for all-cause mortality in patients with CHF and combined sepsis

**Figure 4:**
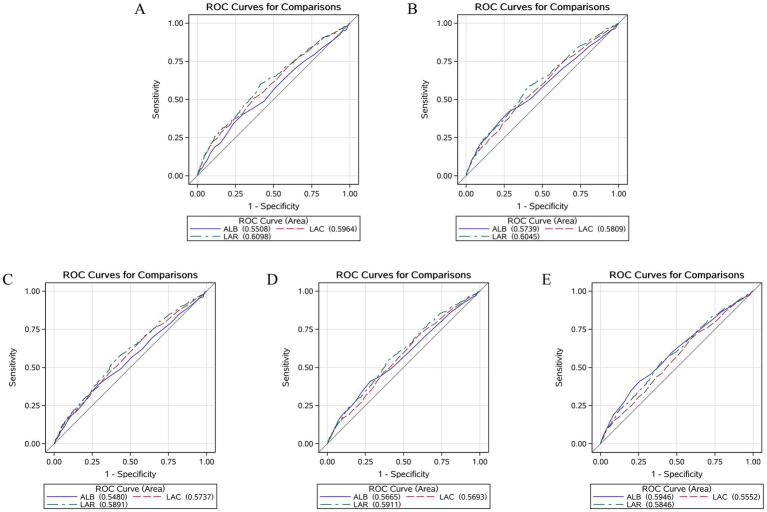
Receiver operating characteristic curves assesses the predictive capability of the LAR for ICU **(A)**, In-hospital **(B)**, 14-day **(C)**, 28-day **(D)**, and 90-day **(E)** all-cause mortality.

We plotted the ROC curves for LAR, lactate, and albumin to evaluate their predictive value for short-term and long-term all-cause mortality in patients with CHF and sepsis. The results showed that LAR’s predictive performance was significantly better than the other two laboratory parameters in predicting ICU all-cause mortality, in-hospital all-cause mortality, as well as 14-day, 28-day, and 90-day all-cause mortality. For detailed results, please refer to Figure 4 and Table 4.

**Table 4:** Information of ROC curves in Figure 4.

Variables	AUC (%)	95% CI (%)	Threshold	Sensitivity	Septicity
ICU mortality					
LAC	59.64	56.56–62.72	2.050	0.630	0.507
ALB	55.08	51.89–58.27	4.150	0.980	0.039
LAR	60.98	59.70–64.07	0.651	0.591	0.596
In hospital mortality					
LAC	58.09	55.44.60.73	2.050	0.630	0.507
ALB	57.39	54.65–60.14	4.150	0.980	0.039
LAR	60.45	57.83–63.07	0.651	0.591	0.596
14-day mortality					
LAC	57.37	54.35–60.38	1.450	0.393	0.714
ALB	54.80	51.69–57.91	4.150	0.979	0.035
LAR	58.91	55.92–61.90	0.651	0.586	0.562
28-day mortality					
LAC	56.93	54.37–59.49	1.450	0.404	0.708
ALB	56.65	54.01–59.29	4.150	0.977	0.026
LAR	59.11	56.57–61.65	0.635	0.586	0.562
90-day mortality					
LAC	55.52	53.18–57.87	1.450	0.412	0.684
ALB	59.46	57.13–61.80	2.750	0.745	0.408
LAR	58.46	56.14–60.79	0.635	0.601	0.537

ROC, Receiver operating characteristic; LAC, Lactate; ALB, Albumin; LAR, Lactate-to-Albumin Ratio.

### 3.5 RCS curve analysis results

**Figure 5:**
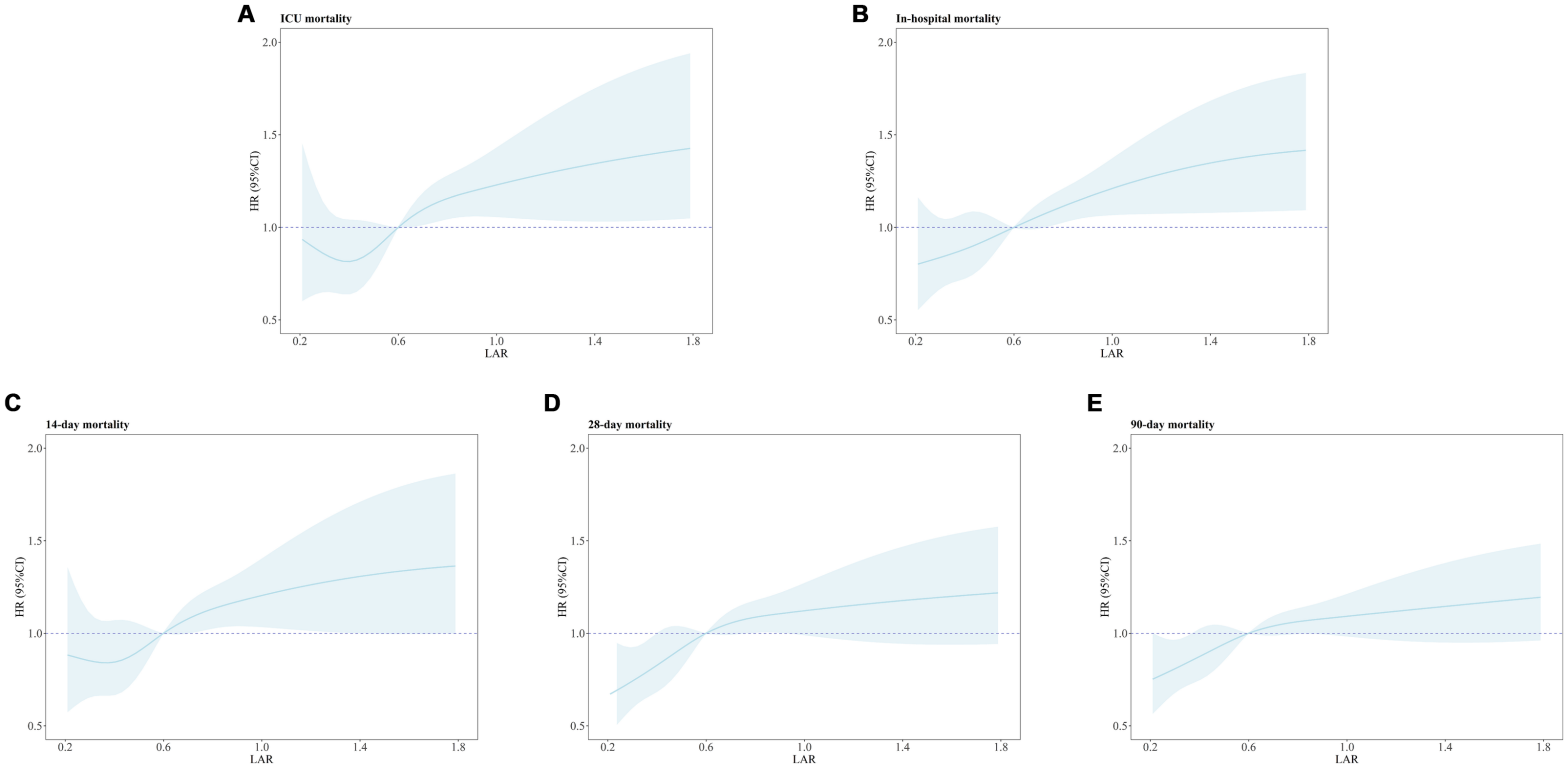
Restricted cubic spline regression analysis of LAR with all-cause mortality. Restricted cubic spline regression analysis of LAR with ICU **(A)**, In-hospital **(B)**, 14-day **(C)**, 28-day **(D)**, and 90-day **(E)** all-cause mortality. ICU mortality: P for overall-linear < 0.001. In-hospital mortality: P for overall-linear < 0.001. 14-day all-cause mortality: P for overall-linear = 0.01.28-day all-cause mortality: P for overall-linear < 0.001.90-day all-cause mortality: P for overall-linear < 0.001.

Figure 5 presents the results of the RCS analysis exploring the relationship between LAR and all-cause mortality at different time points. The RCS analysis showed a significant linear association between LAR and ICU all-cause mortality, in-hospital all-cause mortality, as well as 14-day, 28-day, and 90-day all-cause mortality. Specifically, the *p*-values for the overall linear trend were < 0.001 for ICU all-cause mortality, < 0.001 for in-hospital all-cause mortality, = 0.01 for 14-day all-cause mortality, < 0.001 for 28-day all-cause mortality, and < 0.001 for 90-day all-cause mortality. These results indicate that the linear relationship between LAR and these outcomes is significant. Therefore, an increase in LAR is significantly associated with higher mortality risks at each time point.

### 3.6 Subgroup analysis

**Figure 6:**
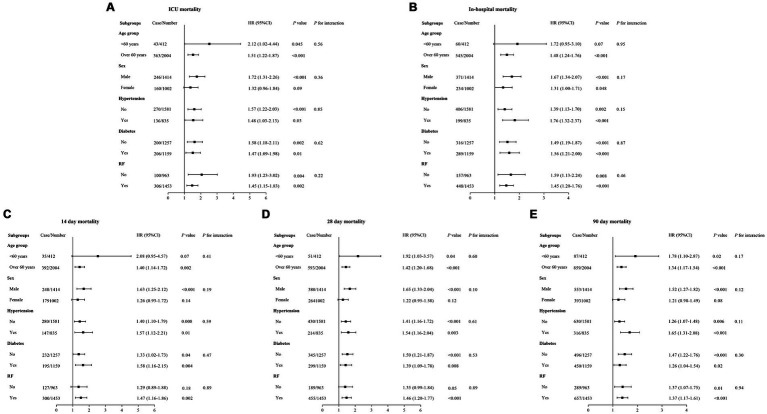
Forest plots of stratified analyses of LAR and ICU **(A)**, In-hospital **(B)**, 14-day **(C)**, 28-day **(D)**, and 90-day **(E)** all-cause mortality.

To further evaluate the prognostic effectiveness of LAR in different clinical subgroups, we conducted a subgroup analysis (Figure 6). The results showed that the prognostic effect of LAR was consistent across all subgroups, and no significant interactions between LAR and these subgroups were observed. This indicates that the effect of LAR as a prognostic marker is consistent across various clinical subgroups, making it applicable to different patient populations. Therefore, LAR holds broad clinical applicability for prognostic assessment in critically ill patients with CHF and sepsis.

## 4 Discussion

This study is the first to systematically evaluate the prognostic value of the LAR in critically ill patients with CHF and sepsis. Through a retrospective analysis of 2,416 patients, we found that an elevated LAR was independently associated with increased ICU, in-hospital, 14-day, 28-day, and 90-day all-cause mortality, and this association was consistent across different clinical subgroups, including age, sex, and other comorbidities. These findings provide a novel perspective on the prognostic assessment of high-risk patients with CHF complicated by sepsis.

CHF is a pathological condition caused by progressive cardiac dysfunction, resulting in inadequate blood circulation. The hallmark of CHF is the heart’s inability to pump blood effectively, leading to poor circulation and systemic hypoperfusion, as well as inadequate organ perfusion. This hypoperfusion state not only causes metabolic disturbances and tissue hypoxia but also leads to immune system dysfunction (26).

Metabolic disturbances and tissue hypoxia are common manifestations in CHF patients (26). Reduced cardiac function leads to decreased blood flow, limiting the delivery of oxygen to tissues, resulting in hypoxia. Under hypoxic conditions, metabolic pathways change, and the accumulation of lactate and other metabolites exacerbates tissue damage (27, 28). Persistent hypoxia further activates inflammatory responses, driving the body into a state of chronic inflammation (29–31). Immunosuppression is also a frequent complication in CHF patients (30). Chronic cardiac dysfunction leads to low perfusion, limiting the function of immune cells such as white blood cells and monocytes, which impairs immune surveillance and reduces the body’s defense against infections and inflammation (32–34). This immunosuppression not only makes patients more susceptible to bacterial, viral, and fungal infections but also leads to rapid disease progression and sepsis once an infection occurs (35).

Moreover, the use of medications such as diuretics, ACE inhibitors, and β-blockers contributes to the immunosuppressive state in CHF patients (36, 37). These drugs, while improving CHF symptoms such as reducing edema, easing the heart’s workload, and enhancing cardiac output, may negatively affect immune function (38, 39). For example, diuretics can lead to electrolyte imbalances, further impairing immune function; ACE inhibitors reduce the generation of angiotensin II, weakening the function of certain immune cells; and β-blockers may inhibit immune responses by affecting sympathetic nervous system activity. The long-term use of these medications in CHF patients weakens their immune defenses, increasing the risk of infections.

Fluid retention and changes in vascular permeability are additional challenges faced by CHF patients (40, 41). Under the pathological state of CHF, patients often experience fluid retention, leading to generalized edema, particularly in the lower limbs, abdomen, and lungs. Fluid retention not only increases the heart’s workload but also changes vascular permeability, providing easier access for pathogens to invade tissues. This alteration in vascular permeability facilitates the entry of bacteria and pathogens, which can spread quickly via the bloodstream and lymphatic system, leading to systemic infections (11, 42, 43).

When CHF patients develop infections, the combination of impaired immune function and systemic hypoperfusion causes rapid progression of the infection, often resulting in sepsis (15). Sepsis is a systemic inflammatory response syndrome triggered by infection, frequently leading to acute organ failure and, in severe cases, death. The occurrence of sepsis exacerbates the patient’s condition and, due to the spread of the inflammatory response, further damages heart function, creating a vicious cycle that significantly increases mortality risk (15).

In the ICU, patients with CHF and sepsis face more complex clinical situations. ICU patients often require invasive treatments such as vascular access, mechanical ventilation, and renal replacement therapy. While these treatments are essential for life support, they also introduce additional risks of infection (44). For instance, the insertion of vascular access devices can lead to venous thrombosis or bloodstream infections, and mechanical ventilation increases the risk of respiratory infections (45). Although the use of broad-spectrum antibiotics helps control infections, overuse may disrupt normal microbial flora and exacerbate immune suppression (46). The use of corticosteroids, while controlling inflammation, can also weaken the immune system’s defenses (47). Renal replacement therapy may cause electrolyte imbalances or alter immune cell function, further increasing the risk of infection (48).

Lactate is an important marker of tissue hypoxia and metabolic disturbances, with elevated levels often indicating a severe stress response (17). The accumulation of lactate is closely associated with systemic inflammation and tissue hypoxia, reflecting the shift toward anaerobic metabolism in cells and tissues. In critically ill patients, particularly those with sepsis, elevated lactate levels not only indicate inadequate tissue perfusion but may also be linked to the activation of endogenous inflammatory responses (49). Elevated lactate levels typically suggest organ dysfunction, such as inadequate perfusion of the kidneys, liver, and heart, which can lead to organ failure (17). Prolonged high lactate levels, particularly in critically ill patients, are strongly associated with poor prognosis and increased mortality risk (49, 50). In contrast, albumin serves as an important indicator of nutritional status, liver function, and systemic inflammation (51). Albumin is synthesized by the liver and plays multiple roles in maintaining plasma oncotic pressure, transporting fatty acids and hormones, and providing antioxidant functions (51). A decrease in albumin levels typically signifies poor nutritional status or liver dysfunction, as well as the exacerbation of systemic inflammatory responses. The reduction of albumin is commonly seen in both acute and chronic inflammatory processes, such as sepsis, liver disease, and kidney disease, and is closely related to the severity of the disease, length of hospitalization, and ultimate prognosis (51, 52).

LAR integrates multiple aspects of the body’s inflammatory response, organ dysfunction, and nutritional status (17). By considering both lactate elevation, which reflects hypoxia and metabolic disturbances, and albumin reduction, which signals immune suppression and malnutrition, LAR offers a more comprehensive assessment of prognosis. Our study further confirms that LAR is significantly associated with mortality risk at various time points in patients with CHF and sepsis. These findings further validate LAR as a potential independent prognostic factor.

LAR, as a simple and readily accessible biomarker, has important clinical applications. By monitoring LAR, clinicians can identify high-risk patients early, assess their metabolic abnormalities, inflammatory response, and nutritional status, and make informed clinical decisions. In ICU and critical care settings, LAR can assist in risk stratification and individualized treatment, guiding therapeutic adjustments and optimizing clinical outcomes. Therefore, LAR has the potential to become an important tool for prognostic assessment in clinical practice, improving the management and prognosis of critically ill patients.

This study has several strengths. First, it is based on a large-scale retrospective analysis with a substantial sample size and comprehensive data. Second, we employed multiple statistical methods (including multivariate Cox regression, Kaplan–Meier survival analysis, and RCS analysis) to investigate the relationship between LAR and prognosis, and subgroup analyses were performed to validate the stability of the results. Finally, the findings provide empirical evidence for the application of novel biomarkers in high-risk CHF patients with sepsis.

This study has several limitations. First, as a single-center retrospective analysis, it is subject to selection and information biases. The MIMIC-IV database, while comprehensive, is derived from a single institution, which may limit the generalizability of our findings. Validation in multicenter prospective cohorts is warranted. Second, due to the structure of the MIMIC-IV database and the nature of ICD coding, we could not fully distinguish chronic heart failure from sepsis-associated cardiomyopathy and subsequent acute heart failure. The lack of time-stamped diagnostic data and echocardiographic parameters prevents precise differentiation, introducing potential diagnostic heterogeneity. Third, although we adjusted for multiple confounders, residual confounding cannot be excluded. Variables such as physician decisions, fluid management, and inotropic use were not available in the dataset. Fourth, we did not assess dynamic changes in LAR during hospitalization. Serial measurements may provide additional prognostic value and merit further investigation. Finally, combining LAR with other biomarkers could enhance predictive accuracy and support individualized risk stratification in patients with CHF and sepsis.

## 5 Conclusion

This study demonstrates that the LAR is a reliable and independent prognostic marker for critically ill patients with CHF complicated by sepsis. Elevated LAR is significantly associated with increased all-cause mortality at multiple time points, including ICU, in-hospital, 14-day, 28-day, and 90-day all-cause mortality, regardless of clinical subgroups. LAR provides a comprehensive assessment of metabolic disturbances, inflammation, and nutritional status, making it a valuable tool for early risk stratification and individualized treatment in this high-risk population. Further validation in multicenter, prospective studies and exploration of LAR’s dynamic changes during hospitalization could enhance its clinical utility, offering improved prognostic accuracy and more refined management strategies for CHF patients with sepsis.

## Data Availability

The original contributions presented in the study are included in the article/supplementary material, further inquiries can be directed to the corresponding author.

## References

[1] Virani SS Alonso A Aparicio HJ Benjamin EJ Bittencourt MS Callaway CW. Heart disease and stroke statistics-2021 update: a report from the American Heart Association. Circulation. (2021) 143:e254–743. doi: 10.1161/CIR.0000000000000950, PMID: 33501848 PMC13036842

[2] Bertero E Maack C. Metabolic remodelling in heart failure. Nat Rev Cardiol. (2018) 15:457–70. doi: 10.1038/s41569-018-0044-6, PMID: 29915254

[3] Ma LY Chen WW Gao RL Liu LS Zhu ML Wang YJ. China cardiovascular diseases report 2018: an updated summary. J Geriatr Cardiol. (2020) 17:1–8. doi: 10.11909/j.issn.1671-5411.2020.01.001, PMID: 32133031 PMC7008101

[4] Soukup J Pliquett RU. Acute kidney injury during sepsis and prognostic role of coexistent chronic heart failure. J Clin Med. (2025) 14:964. doi: 10.3390/jcm14030964, PMID: 39941634 PMC11818498

[5] Che J Song J Long Y Wang C Zheng C Zhou R. Association between the neutrophil-lymphocyte ratio and prognosis of patients admitted to the intensive care unit with chronic heart failure: a retrospective cohort study. Angiology. (2024) 75:786–95. doi: 10.1177/00033197231196174, PMID: 37586709

[6] Jia X Yu XL Lu B Shang YY Shen LF Li YL. Malnutrition and infection lead to poor prognosis and heavy financial burden of patients with chronic heart failure. Front Cardiovasc Med. (2022) 9:1045262. doi: 10.3389/fcvm.2022.1045262, PMID: 36531734 PMC9752848

[7] Myrianthefs PM Lazaris N Venetsanou K Smigadis N Karabatsos E Anastasiou-Nana MI. Immune status evaluation of patients with chronic heart failure. Cytokine. (2007) 37:150–4. doi: 10.1016/j.cyto.2007.03.007, PMID: 17451965

[8] Wilcox CS Testani JM Pitt B. Pathophysiology of diuretic resistance and its implications for the management of chronic heart failure. Hypertension. (2020) 76:1045–54. doi: 10.1161/HYPERTENSIONAHA.120.15205, PMID: 32829662 PMC10683075

[9] Rahman A Jahan N Rahman MT Nishiyama A. Potential impact of non-steroidal mineralocorticoid receptor antagonists in cardiovascular disease. Int J Mol Sci. (2023) 24:1922. doi: 10.3390/ijms24031922, PMID: 36768246 PMC9915890

[10] Palazzuoli A Ruocco G Del Buono MG Pavoncelli S Delcuratolo E Abbate A. The role and application of current pharmacological management in patients with advanced heart failure. Heart Fail Rev. (2024) 29:535–48. doi: 10.1007/s10741-024-10383-0, PMID: 38285236

[11] Gallo G Savoia C. New insights into endothelial dysfunction in cardiometabolic diseases: potential mechanisms and clinical implications. Int J Mol Sci. (2024) 25:2973. doi: 10.3390/ijms25052973, PMID: 38474219 PMC10932073

[12] Mohammadi K Shafie D Ghomashi N Abdolizadeh A Sadeghpour M. Kinin-kallikrein system: new perspectives in heart failure. Heart Fail Rev. (2024) 29:729–37. doi: 10.1007/s10741-024-10393-y, PMID: 38381277

[13] McDonagh TA Metra M Adamo M Gardner RS Baumbach A Böhm M. 2021 ESC guidelines for the diagnosis and treatment of acute and chronic heart failure. Eur Heart J. (2021) 42:3599–726. doi: 10.1093/eurheartj/ehab368, PMID: 34447992

[14] Hemmati M Kashanipoor S Mazaheri P Alibabaei F Babaeizad A Asli S. Importance of gut microbiota metabolites in the development of cardiovascular diseases (CVD). Life Sci. (2023) 329:121947. doi: 10.1016/j.lfs.2023.121947, PMID: 37463653

[15] Liu L Huang P Wang C Liu Y Gao Y Yu K. Causal association between heart failure and sepsis: insights from Mendelian randomization and observational studies. Clin Epidemiol. (2024) 16:755–67. doi: 10.2147/CLEP.S487118, PMID: 39524502 PMC11550685

[16] Hua Y Ding N Jing H Xie Y Wu H Wu Y. Association between the lactate-to-albumin ratio (LAR) index and risk of acute kidney injury in critically ill patients with sepsis: analysis of the MIMIC-IV database. Front Physiol. (2025) 16:1469866. doi: 10.3389/fphys.2025.1469866, PMID: 40046181 PMC11879934

[17] Wang J Chen X Qin C Shi R Huang Y Gong J. Lactate-to-albumin ratio as a potential prognostic predictor in patients with cirrhosis and sepsis: a retrospective cohort study. BMC Infect Dis. (2025) 25:223. doi: 10.1186/s12879-025-10601-6, PMID: 39953385 PMC11829571

[18] Huang T Lin S. Usefulness of lactate to albumin ratio for predicting in-hospital mortality in atrial fibrillation patients admitted to the intensive care unit: a retrospective analysis from MIMIC-IV database. BMC Anesthesiol. (2024) 24:108. doi: 10.1186/s12871-024-02470-4, PMID: 38515077 PMC10956288

[19] Liu Q Zheng HL Wu MM Wang QZ Yan SJ Wang M. Association between lactate-to-albumin ratio and 28-days all-cause mortality in patients with acute pancreatitis: a retrospective analysis of the MIMIC-IV database. Front Immunol. (2022) 13:1076121. doi: 10.3389/fimmu.2022.1076121, PMID: 36591285 PMC9795001

[20] Haas SA Lange T Saugel B Petzoldt M Fuhrmann V Metschke M. Severe hyperlactatemia, lactate clearance and mortality in unselected critically ill patients. Intensive Care Med. (2016) 42:202–10. doi: 10.1007/s00134-015-4127-0, PMID: 26556617

[21] Doenyas-Barak K Beberashvili I Marcus R Efrati S. Lactic acidosis and severe septic shock in metformin users: a cohort study. Crit Care. (2016) 20:10. doi: 10.1186/s13054-015-1180-6, PMID: 26775158 PMC4715304

[22] Artigas A Wernerman J Arroyo V Vincent JL Levy M. Role of albumin in diseases associated with severe systemic inflammation: pathophysiologic and clinical evidence in sepsis and in decompensated cirrhosis. J Crit Care. (2016) 33:62–70. doi: 10.1016/j.jcrc.2015.12.019, PMID: 26831575

[23] Cakir E Turan IO. Lactate/albumin ratio is more effective than lactate or albumin alone in predicting clinical outcomes in intensive care patients with sepsis. Scand J Clin Lab Invest. (2021) 81:225–9. doi: 10.1080/00365513.2021.1901306, PMID: 33745405

[24] Johnson AEW Bulgarelli L Shen L Gayles A Shammout A Horng S. Author correction: MIMIC-IV, a freely accessible electronic health record dataset. Sci Data. (2023) 10:219. doi: 10.1038/s41597-023-02136-9, PMID: 37072428 PMC10113185

[25] Singer M Deutschman CS Seymour CW Shankar-Hari M Annane D Bauer M. The third international consensus definitions for sepsis and septic shock (Sepsis-3). JAMA. (2016) 315:801–10. doi: 10.1001/jama.2016.0287, PMID: 26903338 PMC4968574

[26] Jiang M Fan X Wang Y Sun X. Effects of hypoxia in cardiac metabolic remodeling and heart failure. Exp Cell Res. (2023) 432:113763. doi: 10.1016/j.yexcr.2023.113763, PMID: 37726046

[27] Chen Y Ba J Peng C Peng H Li S Lai W. Impact of lactate/albumin ratio on prognostic outcomes in patients with concomitant heart failure and chronic kidney disease. Intern Emerg Med. (2024) 19:1625–36. doi: 10.1007/s11739-024-03656-x, PMID: 38795274

[28] Nan Tie E Wolsk E Nanayakkara S Vizi D Mariani J Moller JE. Hyperlactataemia is a marker of reduced exercise capacity in heart failure with preserved ejection fraction. ESC Heart Fail. (2024) 11:2557–65. doi: 10.1002/ehf2.14794, PMID: 38698563 PMC11424369

[29] Traub J Beyersdorf N Sell R Frantz S Störk S Stoll G. Plasma levels of sTREM2 in chronic heart failure: predictors and prognostic relevance. Am J Physiol Heart Circ Physiol. (2025) 328:H594–602. doi: 10.1152/ajpheart.00728.2024, PMID: 39918245

[30] Kumar V Bansal SS. Immunological regulation of fibrosis during heart failure: it takes two to tango. Biomol Ther. (2025) 15:58. doi: 10.3390/biom15010058, PMID: 39858452 PMC11763336

[31] Russo I Dun W Mehta S Ahmed S Tzimas C Fukuma N. Extracellular matrix instability and chronic inflammation underlie maladaptive right ventricular pressure overload remodeling and failure in male mice. Am J Physiol Heart Circ Physiol. (2025) 328:H676–92. doi: 10.1152/ajpheart.00331.2024, PMID: 39679492

[32] Amrute JM Luo X Penna V Yang S Yamawaki T Hayat S. Targeting immune-fibroblast cell communication in heart failure. Nature. (2024) 635:423–33. doi: 10.1038/s41586-024-08008-5, PMID: 39443792 PMC12334188

[33] García-Torre A Bueno-García E Moro-García MA López-Martínez R Rioseras B Díaz-Molina B. IL-10 indirectly modulates functional activity of CD4+CD28null T-lymphocytes through LFA-3 and HLA class II inhibition. Immunology. (2024) 173:296–309. doi: 10.1111/imm.13824, PMID: 38922883

[34] Humes HD Aaronson KD Buffington DA Sabbah HN Westover AJ Yessayan LT. Translation of immunomodulatory therapy to treat chronic heart failure: preclinical studies to first in human. PLoS One. (2023) 18:e0273138. doi: 10.1371/journal.pone.0273138, PMID: 37023139 PMC10079025

[35] Fletcher RA Rockenschaub P Neuen BL Walter IJ Conrad N Mizani MA. Contemporary epidemiology of hospitalised heart failure with reduced versus preserved ejection fraction in England: a retrospective, cohort study of whole-population electronic health records. Lancet Public Health. (2024) 9:e871–85. doi: 10.1016/S2468-2667(24)00215-9, PMID: 39486903

[36] Briasoulis A Androulakis E Christophides T Tousoulis D. The role of inflammation and cell death in the pathogenesis, progression and treatment of heart failure. Heart Fail Rev. (2016) 21:169–76. doi: 10.1007/s10741-016-9533-z, PMID: 26872673

[37] Parmley WW. How many medicines do patients with heart failure need? Circulation. (2001) 103:1611–2. doi: 10.1161/01.cir.103.12.1611, PMID: 11273985

[38] Lim AH Abdul Rahim N Zhao J Cheung SYA Lin YW. Cost effectiveness analyses of pharmacological treatments in heart failure. Front Pharmacol. (2022) 13:919974. doi: 10.3389/fphar.2022.919974, PMID: 36133814 PMC9483981

[39] Danielson C Lileikyte G Ouwerkerk W S P Lam C Erlinge D Teng TK. Sex differences in efficacy of pharmacological therapies in heart failure with reduced ejection fraction: a meta-analysis. ESC Heart Fail. (2022) 9:2753–61. doi: 10.1002/ehf2.13974, PMID: 35603531 PMC9288771

[40] Miller WL. Congestion/decongestion in heart failure: what does it mean, how do we assess it, and what are we missing?-is there utility in measuring volume? Heart Fail Rev. (2024) 29:1187–99. doi: 10.1007/s10741-024-10429-3, PMID: 39106007

[41] Maryam Varghese TP B T. Unraveling the complex pathophysiology of heart failure: insights into the role of renin-angiotensin-aldosterone system (RAAS) and sympathetic nervous system (SNS). Curr Probl Cardiol. (2024) 49:102411. doi: 10.1016/j.cpcardiol.2024.102411 38246316

[42] Mohaissen T Proniewski B Targosz-Korecka M Bar A Kij A Bulat K. Temporal relationship between systemic endothelial dysfunction and alterations in erythrocyte function in a murine model of chronic heart failure. Cardiovasc Res. (2022) 118:2610–24. doi: 10.1093/cvr/cvab306, PMID: 34617995 PMC9491865

[43] Suzuki T Suzuki Y Okuda J Kurazumi T Suhara T Ueda T. Sepsis-induced cardiac dysfunction and β-adrenergic blockade therapy for sepsis. J Intensive Care. (2017) 5:22. doi: 10.1186/s40560-017-0215-2, PMID: 28270914 PMC5335779

[44] Kreitmann L Vasseur M Jermoumi S Perche J Richard JC Wallet F. Relationship between immunosuppression and intensive care unit-acquired colonization and infection related to multidrug-resistant bacteria: a prospective multicenter cohort study. Intensive Care Med. (2023) 49:154–65. doi: 10.1007/s00134-022-06954-0, PMID: 36592202

[45] Buetti N Souweine B Mermel L Mimoz O Ruckly S Loiodice A. Obesity and risk of catheter-related infections in the ICU. A post hoc analysis of four large randomized controlled trials. Intensive Care Med. (2021) 47:435–43. doi: 10.1007/s00134-020-06336-4, PMID: 33521871

[46] Hofer U. Antibiotics predispose to nosocomial infections. Nat Rev Microbiol. (2022) 20:445. doi: 10.1038/s41579-022-00756-3, PMID: 35672391

[47] Yang J Chen M Li L Zha Z Cheng M Yang X. Prognosis analysis and infection-related risk factors of multidrug-resistant bacteria isolated from a general hospital in China, 2019-2023. J Hosp Infect. (2025) 158:29–37. doi: 10.1016/j.jhin.2025.02.003 39961512

[48] Montméat V Bonny V Urbina T Missri L Baudel JL Retbi A. Epidemiology and clinical patterns of lung abscesses in ICU: a French multicenter retrospective study. Chest. (2024) 165:48–57. doi: 10.1016/j.chest.2023.08.020, PMID: 37652296

[49] Liu Y. Association between lactate/albumin ratio and 28-day mortality in ICU critical patients with coronary heart disease: a retrospective analysis of the MIMIC-IV database. Front Cardiovasc Med. (2024) 11:1486697. doi: 10.3389/fcvm.2024.1486697, PMID: 39624213 PMC11609210

[50] Duan W Yang F Ling H Li Q Dai X. Association between lactate to hematocrit ratio and 30-day all-cause mortality in patients with sepsis: a retrospective analysis of the medical information Mart for intensive care IV database. Front Med (Lausanne). (2024) 11:1422883. doi: 10.3389/fmed.2024.1422883, PMID: 39193015 PMC11347292

[51] Wang J Yang P Zeng X Chen S Chen X Deng L. Prognostic significance of albumin corrected anion gap in patients with acute pancreatitis: a novel perspective. Sci Rep. (2025) 15:1318. doi: 10.1038/s41598-025-85773-x, PMID: 39779808 PMC11711654

[52] Wang J Li H Luo H Shi R Chen S Hu J. Association between serum creatinine to albumin ratio and short-and long-term all-cause mortality in patients with acute pancreatitis admitted to the intensive care unit: a retrospective analysis based on the MIMIC-IV database. Front Immunol. (2024) 15:1373371. doi: 10.3389/fimmu.2024.1373371, PMID: 38686375 PMC11056558

